# Microbiome-Mediated Protection against Pathogens in Woody Plants

**DOI:** 10.3390/ijms242216118

**Published:** 2023-11-09

**Authors:** Qin Xiong, Jun Yang, Siyi Ni

**Affiliations:** Co-Innovation Center for Sustainable Forestry in Southern China, College of Life Science, Nanjing Forestry University, Nanjing 210037, China; junyang@njfu.edu.cn (J.Y.); siyini@njfu.edu.cn (S.N.)

**Keywords:** microbiome, rhizosphere, phyllosphere, endosphere, plant immunity, pathogen suppression, biological control agents, woody plants

## Abstract

Pathogens, especially invasive species, have caused significant global ecological, economic, and social losses in forests. Plant disease research has traditionally focused on direct interactions between plants and pathogens in an appropriate environment. However, recent research indicates that the microbiome can interact with the plant host and pathogens to modulate plant resistance or pathogen pathogenicity, thereby altering the outcome of plant–pathogen interactions. Thus, this presents new opportunities for studying the microbial management of forest diseases. Compared to parallel studies on human and crop microbiomes, research into the forest tree microbiome and its critical role in forest disease progression has lagged. The rapid development of microbiome sequencing and analysis technologies has resulted in the rapid accumulation of a large body of evidence regarding the association between forest microbiomes and diseases. These data will aid the development of innovative, effective, and environmentally sustainable methods for the microbial management of forest diseases. Herein, we summarize the most recent findings on the dynamic structure and composition of forest tree microbiomes in belowground and aboveground plant tissues (i.e., rhizosphere, endosphere, and phyllosphere), as well as their pleiotropic impact on plant immunity and pathogen pathogenicity, highlighting representative examples of biological control agents used to modulate relevant tree microbiomes. Lastly, we discuss the potential application of forest tree microbiomes in disease control as well as their future prospects and challenges.

## 1. Introduction

Forests offer numerous services to humans, including climate regulation, water supply and regulation, and biodiverse habitats. Nevertheless, the current state of global forest resources does not bode well for their conservation [1]. Apart from human deforestation, outbreaks of well-studied diseases and the emergence of new diseases are some of the most influential factors threatening forest resources. Typical examples of devastating diseases include chestnut ink disease (Dutch elm disease (*Ophiostoma ulmi* and *O. novo-ulmi*) [2], *Phytophthora cinnamomi* and *Phytophthora cambivora*) [3], chestnut blight (*Cryphonectria parasitica*) [4], sudden oak death (*Phytophthora ramorum*) [5], pine wilt disease (*Bursaphelenchus xylophilus*) [6], ash dieback (*Hymenoscyphus fraxineus*), and laurel wilt (*Raffaelea lauricola*) [7]. These diseases have led to the near extinction of forest trees and the degradation of forest ecosystems. The occurrence of forest diseases in nature requires a susceptible host, a causative pathogen, and appropriate environmental conditions (e.g., temperature and humidity) [8,9,10,11], as explicitly stated by the disease triangle theory, which is a widely accepted concept in the field of plant pathology. However, studies have traditionally focused on the interaction between a single woody plant and a single pathogen, neglecting to fully investigate the impact of the plant microbiome on pathogenic colonization and pathogenicity. In the disease triangle, the influence of other woody plant (WP)-colonizing microorganisms, namely the microbiota, on pathogen colonization and plant defense against infection is not considered.

Plant microbiomes exist widely in the rhizosphere, in the phyllosphere, and inside plants. Microbiome-wide association studies (MWAS) have been widely used to investigate the links between the altered microbiome and the development of human diseases, including but not restricted to rheumatoid arthritis, liver cirrhosis, type 2 diabetes, obesity, and colorectal cancer [12]. Due to their present success in the field of human and animal diseases, MWAS have been broadened to investigate the association between the plant microbiome and disease [13,14]. The existing studies suggest that, like the human microbiome, the plant microbiome also plays a significant impact in promoting plant growth and development, facilitating nutrient uptake, and enhancing disease defense [15,16]. Plant microbiomes are not static. Their structure and function change in response to pathogen infection to directly or indirectly regulate the plant’s immune system, ultimately determining plant health. As a result, some related concepts such as the core microbiome [17], synthetic microbiome [18], defense microbiome [19], and agricultural precision microbiome [20] have evolved. Our understanding of the plant microbiome and its role in maintaining plant health has advanced considerably in recent years due to the rapid development of microbiome-related technologies and approaches. The initial description stage has paid much attention to the mechanisms governing the assembly and function of microbiota, and attention has shifted to the potential applications in integrated pest management programs. Key drivers affecting the assembly of plant-associated microbiota are identified through microbiome genome-wide association studies (mGWAS) and metagenomic association studies, which also link certain microbial species to genes involved in plant colonization, plant physiology, and fitness-related features. Metaproteomic and metabolomic technologies have been used to mine and identify key proteins, signaling molecules, hormones, and secondary metabolites in the microbiome, thereby revealing new mechanisms by which the microbiome aids plant defense against disease. In this context, forest pathologists also sense an opportunity to study the relationships between taxonomic composition, the functioning of the WP microbiota, and disease control [21,22,23,24,25].

Strengthening our ability to prevent and control forest diseases and protecting the healthy production of forest trees are major issues facing the development of the forest industry [26]. Trees possess their own unique attributes, such as long generation times, large size, lignification, and complex relationships with other biological populations. As a result, research into the forest tree microbiome has significantly lagged behind that of gramineous and herbaceous plants. Therefore, in-depth and extensive research into forest tree microbiomes will provide new strategies for the introduction of new biological control agents (BCAs) for high-efficiency seedling breeding, disease-resistant breeding, and biological control. This will also contribute to the sustainable production of wood and the ability of forest ecosystems to deal with environmental pressure. In this review, we discuss the ways in which the forest tree microbiome (mainly the rhizosphere and endosphere microbiomes) protects plants against disease, various changes that can occur within the forest tree microbiome upon pathogen infection, and prospects for incorporating tree–pathogen–microbiome interactions into the management of forest health.

## 2. Tree Rhizosphere Microbiome-Mediated Protection against Pathogens

**Figure 1:**
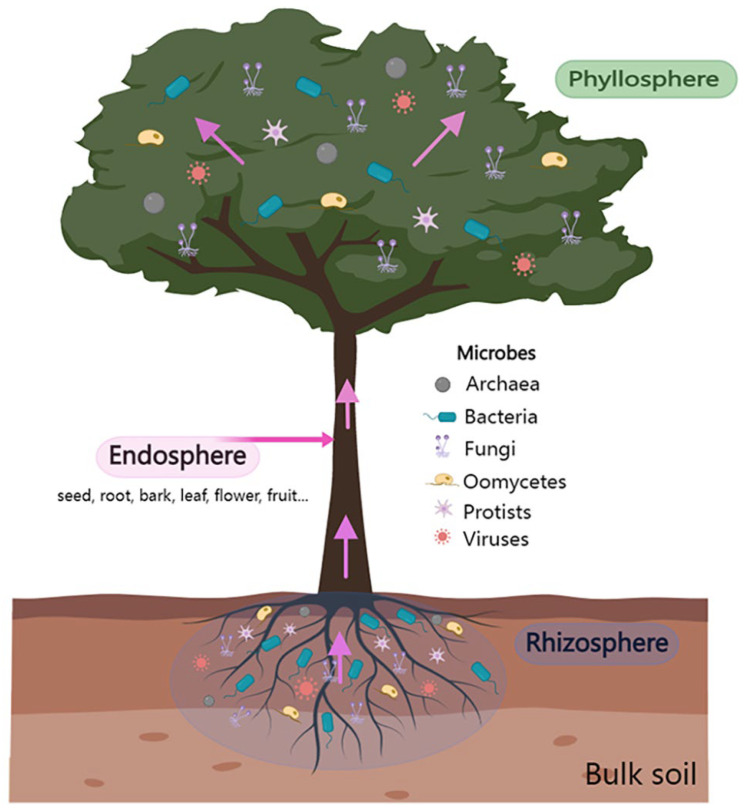
The phyllosphere, endosphere, and rhizosphere are depicted schematically as the major habitats for the diverse microflora that compose plant-associated communities.

In the natural environment, WP–microbiome interactions (in different forms, namely antagonism, amensalism, parasitism, and symbiosis) occur inside (such as in the leaf endosphere and root endosphere) and on WP surfaces (such as in the rhizosphere and phyllosphere) [27,28] (Figure 1). The above- and belowground microbial communities are actively recruited vertically via seeds and pollen, as well as horizontally via soil, atmosphere, and insects [29] to promote plant growth and development, enhance stress tolerance, and improve disease resistance. Plant roots provide a unique niche for soil microbial communities, attracting a wide variety of microbial communities that are then distributed in the rhizosphere, in the roots, and on the surface. Among them, the rhizosphere is a hotspot of microbial habitats and activities and is an important location for energy flow and material exchange between plant roots and soil [30,31]. The selective recruitment of microorganisms from bulk soil and their interrelated systems is called the rhizosphere microbiome (Figure 1). The interaction between the rhizosphere microbiome and a host plant expands the functional scope of the host plant.

### 2.1. Soil-Borne Pathogens and Beneficial Microbes in Tree Rhizosphere

Compared to herbaceous and cereal plants, the rhizosphere zone of woody plants is larger, contains more microorganisms, has increased variety, and is more active. When certain microorganisms expand and reproduce in large numbers, they have the potential to cause root diseases in forest trees. For example, *P. cinnamomi* is an extremely damaging and prevalent soil-borne pathogen that infects several woody plant hosts, such as eucalyptus, oaks, chestnuts, and pines [32]. *Ralstonia solanacearum* causes bacterial wilt in eucalyptus [33] and fig trees [34]. *Ceratocystis* sp. causes damping off in many forestry species, including *Acacia* spp., *Eucalyptus* spp., and *Quercus* spp. [35,36]. Vascular wilt is caused by the fungus *Verticillium dahliae*, which is a classical soil-borne disease that presents a threat to olive trees [37], smoke trees [38], and many other tree hosts [39].

Approximately 2–5% of rhizosphere microorganisms mainly form two types of symbioses, arbuscular mycorrhiza (AM) and ectomycorrhiza (ECM), with forest roots, which have a significant impact on promoting plant growth and reducing plant diseases [40,41,42,43]. Some can be used as biocontrol agents (bacteria: *Bacillus* and *Pseudomonas*, fungi: *Trichoderma* and yeast, and actinomycetes: *Streptomyces*) to inhibit pathogen growth and reproduction by competing with pathogens for rhizosphere nutrition and niches, or by secreting siderophile elements, hydrolytic enzymes, volatile organic compounds, and antibiotics, thereby indirectly promoting plant growth [21,44]. For instance, the *Pseudomonas fluorescens* strain PICF7 has been applied to olive trees to induce host resistance to Verticillium wilts caused by the pathogen *V. dahliae* [45,46]. Some plant growth-promoting rhizobacteria (PGPR) can function as biological triggers to induce resistance as well as decrease plant disease severity and incidence under greenhouse and field conditions [47].

### 2.2. Root Exudates and Microbial Signal Molecules Affecting Rhizosphere Microbiome of Trees

Trees can change the composition of rhizosphere microbiota by secreting bioactive exudates (e.g., organic acids, fatty acids, amino acids, phenolics, nucleotides, plant growth regulators, sterols, sugars, vitamins, and putrescine) into the region of the rhizosphere [48,49,50,51]. Root exudates, in addition to serving as sources of carbon and nitrogen substrates for microbial proliferation, have many other effects on rhizosphere microbes by functioning as signaling molecules, stimulants, attractants, inhibitors, or repellents [52,53]. For example, Rudrappa et al. [54] demonstrated that L-malic acid, an intermediate product of the tricarboxylic acid cycle that is secreted from *Arabidopsis* roots, selectively signals and, in a dose-dependent manner, recruits the beneficial rhizobacterium *Bacillus subtilis*, resulting in a stronger immune response to *Pseudomonas syringae* pathovar *tomato* (*Pst*). Salicylic acid (SA), a defense phytohormone secreted in the *Arabidopsis* root zone, has been reported by Lebeis et al. [55] to modulate root microbiome colonization via particular bacterial taxa, thereby contributing to the root microbiome composition. Banana root exudates contain fumaric acid, which attracts *B. subtilis* N11 and promotes biofilm formation [56]. The roots of the Chinese tallow tree (*Triadica sebifera*) release flavonoids that can help AM fungal spores germinate and colonize. These flavonoids may also have an effect on soil microbes and other soil processes [57].

Since the types and concentrations of root exudates vary among different plant species and among different natural populations within the same plant species, and there are different plant growth stages, nutritional statuses, and stress exposures [50,58,59], the host genotype has a significant impact on the composition of the rhizosphere microbiome [60]. In addition, rhizosphere microbes can produce and release signaling molecules (e.g., volatile organic compounds, oxalic acid, and glucose) or extracellular metabolites (e.g., amino acids and vitamins), which can change both microbe-to-microbe and plant-to-microbe communication in the rhizosphere. For example, fungal hyphae are used by bacteria for migration into soils in an interaction known as “fungal highways”. This process is a synergistic example of inter-microbial assisted dispersal [61,62]. Further, *N*-acyl-homoserine lactone-driven quorum-sensing (QS) signaling molecules are produced by PGPRs, which are responsible for promoting plant growth [63] and inducing defense against pathogens [64]. Microbial volatile organic substances (VOCs), including 2,3-butanediol, 3-phenyproprionic acid, chokol K, and 3-octanone, function as chemical warfare against other microbes, as regulators of plant development and stress resistance, and as signals that mediate intra- and interspecies communications [65,66,67].

### 2.3. Root Microbiome Enhances Plant Disease Resistance

The interactions between plants and the rhizosphere microbiome can generate a strong selective pressure that shapes the rate and pattern of microbial evolution and eventually affects the rhizosphere microbiome’s composition [68]. In response to pathogen attack, plants can send specific signals that directly hinder pathogen growth and invasion or encourage the recruitment of beneficial microbes that are able to improve plant defense responses. This phenomenon is commonly referred to as the “cry for help hypothesis” [69,70,71].

In comparison to annual and/or herbaceous plants, perennial woody plants can establish long-term associations with their associated microbiome. For most crops used in previous studies, short growth periods limit the stability of the rhizosphere microbiome. Therefore, their role in plant resistance is readily disrupted by environmental factors [72,73]. As a result, it is difficult to gain additional insights into the importance of plant species selection and the role that environmental filtering plays in the composition of the rhizosphere microbiome in periods of short-term growth. Conversely, due to long-term interactions between trees and the soil microbiome, trees can establish relatively stable rhizosphere microbiome characteristics and plant functional traits [52,74], which enhance biotic stress tolerance against soil-borne pathogens. Taken together, optimizing the tree rhizosphere microbiome may be an effective way to improve forest health [75,76,77].

## 3. Tree Phyllosphere Microbiome-Mediated Protection against Pathogens

The phyllosphere (all aboveground compartments of a plant, often leaves) accounts for a substantially larger proportion of a terrestrial plant than the root system. Leaves, with a global leaf envelope estimated to be about one billion km^2^ [78], represent one of the richest habitats on earth [79] and are inhabited on the outside (i.e., epiphytic) and inside (i.e., endophytic) by a diverse range of microbes, primarily bacteria, filamentous fungi, and yeast strains [80], followed by protists [81] and bacteriophages [82] (Figure 1). However, in comparison to those of rhizosphere microbes, the functional roles of phyllosphere microbes remain a subject that has been less explored. Emerging evidence suggests that the phyllosphere microbiome is also recognized as a significant contributor to plant health and growth [80,83]. Yet, there is limited understanding of the underlying mechanism through which the phyllosphere microbiome engages with the host immune system and consequently facilitates the development and defense of the host plant.

### 3.1. Multiple Factors Drive the Colonization of Microbial Aggregates on the Phylloplane

The colonization source of the phyllosphere remains somewhat uncertain. Recent research indicates that soil is the ultimate source of phylloplane microorganisms [84]. At the same time, a wealth of studies have been conducted to investigate the impact of soil and rhizosphere bacterial communities on the colonization of phyllosphere bacteria in *Arabidopsis thaliana* [85,86,87,88] and maize [89]. Bacteria are the most abundant and diverse microbial group in the phyllosphere [79], but their diversity is comparatively lower when compared to that observed in the rhizosphere or soil [90,91]. Izhaki et al. [92] identified 32 bacterial species associated with *Citrus paradisi* leaf surfaces. Epiphytic mycobiota on the phylloplane of two deciduous trees and three evergreen shrubs increases during plant growth [93]. In characterizing phyllosphere bacterial communities in a neotropical forest, Kembel et al. [94] showed that the leaves of individual trees served as habitats for more than 400 bacterial taxa, and a core microbiome of taxa, including *Actinobacteria*, *Alpha-*, *Beta-*, and *Gammaproteobacteria*, as well as *Sphingobacteria*, dominated these phyllosphere bacterial communities. Generally, the bacterial phyla belonging to Proteobacteria, *Firmicutes*, *Actinobacteria*, and *Bacteroidetes* are found to be the most abundant in several plant phyllospheres, including *A. thaliana*, common ash (*Fraxinus excelsior*), poplar, and other tree leaves [85,87,94,95,96,97,98].

Usually, soil types and properties, the climate, genetic characteristics of plants (genotype and phenotype), geographical location, and attack by pathogens, among other factors, are drivers that contribute to the assembly of phyllosphere bacterial communities [99,100,101]. However, the mechanism underlying how these factors regulate microbial communities in the phyllosphere is not fully understood. Moreover, there is still controversy about the main determining drivers. Finkel et al. [102] found that different species of the salt-excreting desert *Tamarix* trees (*T. nilotica*, *T. aphylla*, and *T. tetragina*) cultivated in the same geographical location possessed strikingly similar bacterial communities, suggesting that the major determinant of the composition of phyllosphere bacterial communities was geographical location, not plant species. In a later study, sampling of leaves from *T. aphylla* trees growing across the Sonoran Desert in the southwestern United States provided additional evidence of the main impact of geographic distances on phyllosphere bacterial communities [103]. However, some other studies offered contradictory insights, showing that the genetic traits of plants drive the bacterial community composition on leaf surfaces. For example, regardless of the geographical location where the leaf samples were obtained, the phyllosphere bacterial communities associated with *Pinus ponderosa* exhibited remarkable similarity among them. Additionally, leaves collected from 56 distinct tree species originating from the same geographical location contained bacterial populations unique to each plant species [98]. In a study examining the leaves of five tree species, Laforest-Lapointe et al. [104] also indicated that host species identity is a more crucial factor contributing to the phyllosphere bacterial communities of temperate trees compared to forest sites or time.

The fungal microbiota of the phyllosphere consists of two dominant groups: yeast and filamentous fungi [105]. On healthy plant leaves, yeasts are the main foliar epiphytic fungi. Because they contain many pathogens of plant systems, phyllosphere fungi have received a lot of attention. Infectious filamentous fungi must survive in a harsh and unstable phyllosphere environment, such as one with nutrient deprivation, high UV radiation, and high temperature and humidity fluctuations [105], and need to escape from various direct and indirect physical damages until the conditions become suitable for plant infestation. The filamentous fungal communities in the phyllosphere are highly diverse at all stages of plant growth, including a variety of pathogenic and non-pathogenic fungi such as *Alternaria*, *Fusarium*, *Cladosporium*, *Penicillium*, *Mucor*, *Colletotrichum*, *Acremonium*, and *Aspergillus* [93,106,107]. In response to global warming, the beneficial microbiota in the phyllosphere has been shown to decrease, whereas there is an enrichment in pathogens [108,109]. At present, the main drivers of phyllosphere fungal communities remain difficult to summarize across different plant species. For instance, soil is the main driver in determining the composition, diversity, and abundance of fungal communities on oak (*Quercus macrocarpa*) leaves [110]. On beech (*Fagus sylvatica*) leaves, fungal community assembly is determined by host genetics [105]. Similarly, the host genotype is the most influential factor determining the assembly of the foliar fungal microbiome of balsam poplar (*Populus balsamifera*) [111,112]. In general, the composition of phyllosphere microorganisms is the combined result of flora competition, climate selection, and host selection [98,113,114,115,116,117].

### 3.2. Stability of Microbial Consortia against Pathogen Perturbation

The leaf surface creates stressful and unstable conditions, including deficiency in nutrient supplies, harmful ultraviolet (UV) radiation, oxidative stress, low water content, fluctuating temperature throughout the day, and biotic stress. Phyllosphere-associated microbes, regardless of whether they are commensal, beneficial, or detrimental microorganisms, employ distinct fitness strategies (tolerance or avoidance strategies) to deal with this combination of stresses for successful colonization [118]. For example, nonpathogenic phyllosphere-colonizing bacteria actively affect their host plant and gain fitness advantages by secreting biosurfactants, modulating plant hormone mimics (i.e., cytokinins and auxins), and mediating plant–pathogen interactions [80,119,120]. Finally, some of these host-adapted microbial colonizers, the epiphytes, remain on the surface of plant organs, while others, the endophytes, can penetrate deeper into plants [121]. The stability of leaf-associated microbial communities is known to be closely related to plant health. Recent studies have also revealed that plants manipulate the phyllosphere microbiota to maintain plant health. According to Chen et al. [122], the *Arabidopsis mfec* mutant and *cad1* mutant displayed leaf damage (chlorosis and/or necrosis phenotypes) and simultaneously caused marked changes in the endophytic phyllosphere microbiota of *A. thaliana*, including an overall increased bacterial population size, decreased community diversity, and a shift from a Firmicutes-rich community to a Proteobacteria-rich community. To the best of our knowledge, this is the first report regarding the genetic regulation of microbiome assembly in the phyllosphere and its consequential impact on plant health. However, significant knowledge gaps still exist about this aspect.

### 3.3. Phyllosphere-Mediated Resistance to Pathogen Invasion

Plants can utilize the “cry for help” strategy whereby they selectively recruit beneficial microbes from the surrounding environment to assist in combating pathogen stress [123,124,125]. Such plant–microbe interactions confer a protective effect on plant health [126]. Not only do rhizosphere microbiome responses contribute significantly to pathogen resistance and plant health [71,127,128], so does the phyllosphere microbiome. For example, Berg and Koskella [129] found that the phyllosphere microbiota of tomato plants conferred protection against *Pst*, which is the causal agent of bacterial speck. Furthermore, the degree of protection was determined by the initial dose of the microbiota administered. Similarly, considerable changes in leaf-associated fungal and bacterial communities were noted upon Huanglongbing (HLB) infection in citrus [130] and upon powdery mildew infection in oak (*Quercus robur*) [131], respectively. The Jakuschkin study further investigated the pathobiontic network of distinct fungal and bacterial operational taxonomic units that were connected with each other and engaged in direct interaction with *Erysiphe alphitoides*, suggesting that some of these taxonomic units potentially conferred protection upon the oak phyllosphere by inhibiting *E. alphitoides*, thereby reducing the incidence of powdery mildew. More recently, Li, Zhu, Zhang, Xu, Wang, Wang, and Li [69] reported that the phyllosphere microbiota of diseased citrus leaves displayed a more intense microbial network and larger numbers of indigenous microbes (e.g., *Sphingomonas* spp.) and newly recruited microbes (e.g., *Methylobacterium* asv41 and *Pantoea* asv90) compared to those of uninfected leaves by the melanose pathogen *D. citri*. Further, *Sphingomonas* spp. exhibited promising results in protecting the citrus phyllosphere against disease invasion through its ability to compete for iron. Overall, the roles played by phyllosphere microbiomes in conferring resistance against pathogen invasion and their contribution to plant health have only recently gained attention and provided a significant opportunity to develop microbiome-based tools for disease prevention or prediction.

## 4. Contribution of Tree Endosphere Microbiome in the Control of Forest Diseases

Endophytes that colonize within plants can confer ecological advantages to the host plants and improve plant fitness, (i.e., improving nutrient uptake, promoting growth and development, imparting abiotic stress tolerance, and providing pathogen resistance) [132]. Endophytes have attracted attention in relation to their capacity to improve disease resistance in *Poaceae* Barnhart and *Brassicaceae* Burnett plants compared to forest plants. Endophytes secrete specialized metabolites or bioactive compounds that help keep plants away from pathogens by means of antagonism, mycoparasitism, and induction of plant defense responses. For example, endophytic bacteria produce defensive enzymes (e.g., polyphenol oxidase, β-1,3-glucanase, chitinase, and peroxidase) [133,134] and phytohormones (ethylene, indole acetic acid, SA, abscisic acid, and jasmonic acid (JA)) [135], and secondary metabolites (phenols, antibiotic, and fungicides) [136,137,138,139] to enable plants to establish robust resistance against pathogens.

Endophytes may promote the induction of defense-related genes and confer pathogen resistance [140]. For instance, the fungal leaf endophyte *Colletotrichum tropicale* induces the expression of hundreds of host defense-related genes in *Theobroma cacao*, leading to enhanced plant immunity [141]. Moreover, *Phoma medicaginis*, which causes alfalfa leaf spots, is resisted by arbuscular mycorrhizal fungus through the activation of defense activities, including JA, SA, peroxidase, and polyphenol oxidase [142]. Pathogen-induced activation of enzyme-coding genes associated with fungal cell wall degradation and biosynthetic gene clusters encoding polyketide synthases and non-ribosomal peptide synthases are responsible for disease-suppressive functions in endophytes [71]. The endophytic microbiome can provide an extra layer of defense by selectively enriching microbes with genetic machinery that produces enzymes and secondary metabolites against pathogens [71,76].

Relatively few studies have been conducted on the utilization of biocontrol endophytes for the purpose of managing forest pests and diseases. Most of the available studies have largely focused on identifying beneficial endophytes that antagonize pathogens and structurally characterizing their metabolic antimicrobial compounds [143,144]. For example, endophytes that Sharon Doty’s lab previously isolated from poplar and willow plants exhibited biocontrol activities against extremely virulent plant pathogens (*Fusarium culmorum*, *Rhizoctonia solani*, *Pythium ultimum*, and *Gaemannomyces graminis* var. *tritici*) as well as promoting plant growth [145,146]. Examples of bioactive natural compounds identified from beneficial endophytes of woody perennials included phomopsolides and the stable α-pyrone isolated from the conifer endophyte *Diaporthe maritima*, which exhibited antifungal activity against *Microbotryum violaceum* and *B. subtilis* [147]. In addition, five foliar fungal endophytes isolated from *Pinus strobus* (eastern white pine) were found to produce potently antifungal compounds, as reported by Sumarah et al. [148]. Further investigation revealed that pyrenophorol in the culture filtrates of the pine endophyte *Lophodermium nitens* was accountable for suppressing the growth of the pine pathogen *Cronartium ribicola* [149]. Other bioactive antimicrobial metabolites (lipopeptides, pyrrolizin, glucoamylase, garcinia cambogia chlorophyll, and chitinase) were also shown to inhibit or kill pathogens, which alleviated forest tree diseases [150]. Thus, the capacity of endophytes to alter plants’ interactions with pathogens may provide integrated disease management tools. However, the specific mechanisms underlying endophyte-mediated pathogen resistance in the aforementioned studies are not fully understood.

## 5. Pathogen Invasion Triggers Innate Immune Responses in Plants

Plants make good use of two types of immune receptors that are located on the cell surface and inside of cells which can recognize invasive microorganisms (e.g., fungi, oomycetes, bacteria, viruses, nematodes, and insects) and activate protective immune responses in plants. However, when pathogens infect host plants, they secrete effectors into the host cells or extracellular space to interfere with host physiological activities to facilitate their infection or colonization. Cell surface immune receptors, also known as “pattern-recognition receptors (PRRs)”, comprise mainly single transmembrane receptor-like kinases and receptor-like proteins that detect pathogen-associated molecular patterns (PAMPs), such as bacterial flagellins, bacterial elongation factors, fungal chitin polysaccharides, fungal xylanases, and endogenous elicitors. The immunity triggered by PRRs is known as pattern-triggered immunity (PTI). Intracellular immune receptors are mainly a class of receptor proteins containing nucleotide-binding and nucleotide-binding leucine-rich repeat receptors, which recognize cytoplasmic effector proteins to elicit effector-triggered immunity.

In addition to pathogens, some plant symbiotic microbes also possess microbe-associated molecular patterns (MAMPs) that can stimulate the innate immune response (MAMP-triggered immunity, MTI), which is similar to PTI [151]. When symbiotic microbes are present, host plants will actively reduce PRR expression levels and MAMP responsiveness to allow symbiotic microbes to thrive and exert their beneficial effects [151,152]. For example, in mycorrhizal symbiosis, the mycorrhizal symbiotic receptor, OsMYR1, and its ligand, CO4, competitively bind OsCERK1, thus inhibiting the formation of immune receptor complexes between OsCERK1 and OsCEBiP, which weakens PAMP-mediated immune responses to promote its own symbiosis [153]. Therefore, the immune system may also be an alternative route to affect the plant microbiome.

## 6. The Microbiome Enhances Plant Immunity and Functions as an Extension of the Plant Immune System

**Figure 2:**
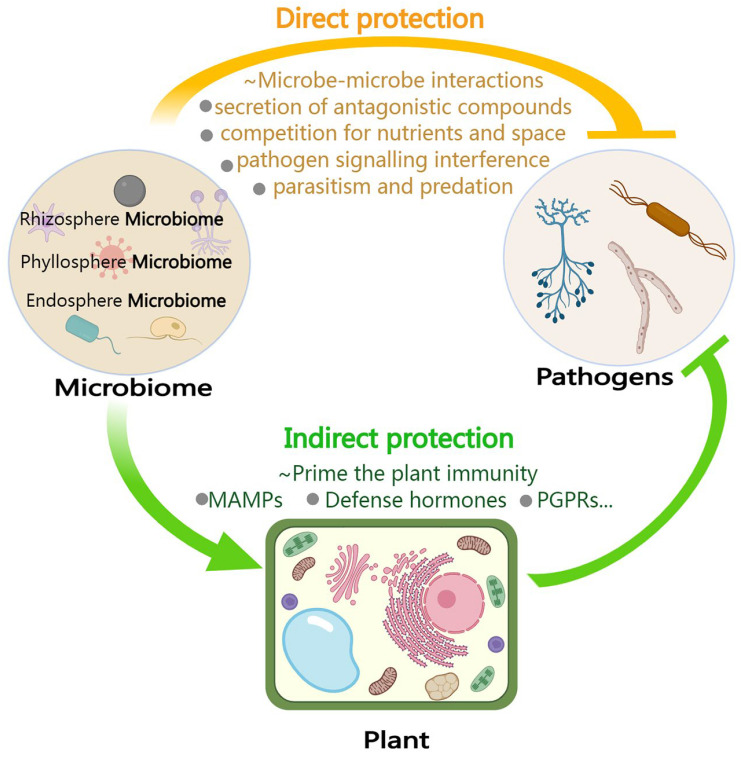
Both interactions of the microbiome with the host and pathogens contribute to plant protection against pathogens. Plants infected by pathogens can recruit and enrich beneficial microbial communities to directly antagonize the pathogens via a variety of methods, such as the “cry for help” strategy. Specific mechanisms include secretion of antagonistic compounds (such as antibiotics, lysozyme, and volatile substances), competition with pathogens for ecological niche and nutrients, inhibition of pathogens’ signaling systems, parasitism, and predation, and so on, thus directly inhibiting pathogen proliferation and infection. On the other hand, the plant microbiome can also act indirectly by priming plant immunity, such as induced systemic resistance (ISR) triggered by microbe-associated molecular patterns (MAMPs), defense hormones, PGPRs, and so on, to inhibit pathogen invasion and protect the underground and aboveground parts from encroachment.

Increasing evidence indicates that under pathogen stress, the composition of the plant microbiome changes, leading to plants actively recruiting beneficial or protective microbes [154] to aid pathogen resistance and improve plant immunity [100,154,155,156]. Recently, it was demonstrated that the infection of citrus trees by *Candidatus Liberibacter* asiaticus, associated with HLB, drastically altered the composition of citrus microbiome communities across the disease spectrum [130]. Moreover, specific compounds present in tree root exudates stimulate key rhizosphere bacteria to collaborate in the suppression of soil-borne pathogens [77]. The plant microbiome interacts with pathogens directly or indirectly to strengthen pathogen resistance (Figure 2).

### 6.1. Acting Directly against Pathogens: Direct Interaction between Microbes

Pathogen-infected plants can recruit and enrich beneficial microbial communities to directly antagonize infecting pathogens via a variety of methods, such as the “cry for help” strategy. Specific mechanisms include competing with pathogens for ecological niches and nutrients, secretion of antagonistic compounds (such as antibiotics, lysozyme, and volatile substances), parasitism and predation, and inhibition of pathogen signaling systems, among others (Figure 2). For example, *Pantoea agglomerans*, a Gram-negative bacterium, exhibits antibiosis activity that limits the growth of *Erwinia amylovora*, which is the causative agent of fire blight disease on apple and pear trees [157,158]. *Bacillus* sp. strains secrete lipopeptides (like Surfactin, Iturin, and Fengycin), polyketides, surfactants, and VOCs, among others, to directly kill or suppress pathogen growth, thereby improving the natural soil resistance against pathogens [159,160,161,162]. Some probiotic *Bacillus* bacteria can also eliminate pathogens by interfering with pathogen QS signaling [163]. Beneficial fungi can effectively inhibit the infection of destructive fungal pathogens such as *Sclerotinia sclerotiorum*, *Fusarium oxysporum*, and *Rhizoctonia solani* through mechanisms of parasitism [164,165,166]. Therefore, the plant microbiome offers a goldmine of antagonists that can directly inhibit pathogens.

### 6.2. Acting Indirectly against Pathogens: Stimulating or Inducing Plant Immunity

The plant microbiome can act indirectly by priming plant immunity (e.g., ISR) to inhibit pathogen invasion and protect the under- and aboveground parts from encroachment (Figure 2). For example, a disease-resistant synthetic bacterial community was constructed in a previous study and found to protect plants by means of a synergistic mechanism, wherein high-abundance bacteria suppress fungal pathogen growth and low-abundance bacteria stimulate plant ISRs [167]. Foliar infection by the downy mildew pathogen *Hyaloperonospora arabidopsidis* leads to the promotion of three bacterial species (*Stenotrophomonas*, *Xanthomonas*, and *Microbacterium*) in the rhizosphere of *Arabidopsis* plants, conferring a soil-mediated legacy that provides enhanced resistance against this pathogen [168]. A set of important studies have provided a wealth of data showing that infection with pathogens in the above- and belowground plant tissues results in a change in the pattern of root secretions, thereby inducing the selective recruitment of root-associated beneficial microbes and forming soil memory or legacy effects of disease resistance to defend subsequent populations of plants growing in the same soil from the same pathogen [70,168,169]. In general, the recruitment of beneficial microbes in the plant rhizosphere during pathogen invasion is beneficial to better mobilize the role of the rhizosphere microbiome in the plant defense system, enhance the ability to control plant diseases, and even unlock new breeding strategies. However, further work is required to understand the mechanisms by which plants recruit beneficial microbes to resist disease and how the microbiome initiates ISR.

## 7. Future Perspectives: Integrating Tree–Pathogen–Microbiome Interactions into Forest Pest Management

### 7.1. The Challenges of Applying BCAs in Protecting Trees

Compared to annual crops like wheat, potatoes, or herbaceous plants, tree generations can persist for decades. Therefore, strategies to control tree diseases need to be effective over many years, not just during a specific growing season [170]. In plantations, the long lifespan and high economic costs of trees do not always allow for regular switching of cultivated species (e.g., crop rotation). In addition to these limitations in the application of agronomic and cultural practices, the application of chemical treatments raises concerns over environmental contamination and adverse effects of chemicals on both human and animal health. Thus, manipulation of the plant microbiome is widely regarded as a highly promising and environmentally friendly strategy for enhancing plant defense against pests.

In recent years, the potential for BCAs has been realized as a crucial part of integrated pest management. The present study builds upon the advancements of omics approaches, synthetic communities, and microbial network inference to construct beneficial microbial consortiums with the potential to support broad and persistent plant protection. Such strategies are also increasingly being adopted in planted forests, despite the fact that just a few instances of success are known. Importantly, the adaptation of endophytes to plant interior tissues and their persistence within plants for long periods of time make endophytes potentially promising as biocontrol agents for chronic woody plant diseases [171]. In fact, tree endophytes have been suggested as a valuable means of sustaining forest health [172]. Effective and/or promising uses of endophytes (bacterial and fungal endophytes) to control tree diseases are currently being implemented.

### 7.2. The Advantages in Tree Pathogen Inhibition by Multi-Strain BCAs over Single-Strain BCAs

In several cases in the management of soil-borne crop diseases, various BCA consortia comprising two or more microbial strains appear to possess enhanced biocontrol activity compared to single-strain BCAs [18,173,174,175]. There is increasing interest in the utilization of multi-strain BCAs for controlling forest diseases. A consortium consisting of *Bacillus velezensis* Bs006 and *Trichoderma virens* GI006 was found to be more efficient against infection by *Fusarium* wilt in cape gooseberry than individual strains [176]. A bacterial consortium of *Trichoderma harzianum* CBF2 and *Pseudomonas aeruginosa* DRB1conferred more consistent protection against *Fusarium* wilt in banana than individual community members [177]. The interactions among the members of multi-strain BCAs might lead to their superior stability and efficiency in improving disease-suppressing effects. Microbial interactions occurring within the plant microbiome play a crucial role as selective forces that help to establish complex microbial assemblages [178]. Consequently, when constructing multi-strain BCAs, it is important to focus on the elements related to the microbe−microbe interplay.

### 7.3. Microbial Interactions Promote Rhizosphere Colonization

A large number of microbe−microbe interactions have been documented to have a positive impact on facilitating rhizosphere colonization by beneficial microorganisms through boosting biofilm formation, microbial proliferation, migration within the microbiome, and interaction with plant roots. For examples, a five-species biocontrol community was found to lead to a higher formation of biofilm compared to single strains, both in vitro and in vivo, thus stimulating host root colonization [179]. *Hyphomicrobium* spp. is capable of associating with methanotrophs, therefore establishing a microbial association in the rhizosphere. This association enables *Hyphomicrobium* spp. to eliminate methanol, thereby preventing methanotrophs from proliferating in the rhizosphere [180]. The syntrophic interactions between multiple microorganisms can promote the proliferation of rhizosphere microbes by removing harmful substances; fungal hyphae, also referred to as “fungal highway”, can serve as vectors for the dispersal of bacteria in the rhizosphere [61,62], indicating that the migration of microorganisms can be facilitated by their interactions within the community. Thus, the application of multi-strain BCAs with active interactions among their members has the potential to increase the survival of disease-suppressing microbes, as well as their adaption to challenging and shifting environmental conditions. As a result, they may possess the capacity to maintain their advantageous impacts in inhibiting forest diseases.

### 7.4. Microbial Interactions Suppress Forest Pathogen Growth

Multi-strain BCAs have been found to provide enhanced suppressive efficacy against pathogen growth compared to single-strain BCAs. This can be attributed to the beneficial effects of inter- and/or intra-domain interactions within multi-strain BCAs, including the increased capability to utilize resources [181,182,183,184] and the biosynthesis of antimicrobial compounds [185,186], which contribute to strengthening the inhibition of pathogen growth.

### 7.5. Microbial Interactions Induce Enhanced Plant Defense Responses against Pathogens

In addition to the above positive features in controlling diseases via the application of multi-strain BCAs, inducing elevated plant defense against plant pathogens through biocontrol consortia has been described in many studies. This is mainly directed by activating a number of distinctive metabolic and signaling pathways in order to combat a given disease [174,187,188]. However, further research is still needed to be better understand how interactions among multi-strain BCA members may successfully improve specific systemic resistance to pathogens. One possibility is that the interactions between microbes inside biocontrol consortia may result in the generation of certain elicitors and potent compounds that can more efficiently elicit ISR, as already discussed in Section 5.

### 7.6. Future Perspectives on Applying BCAs in Protecting Trees

Because of the inherent characteristics of trees (e.g., complicated anatomy, large biomass, perennial nature, and longevity) and pathogens (e.g., complex life cycles and complex infection), the implementation of biological control strategies in trees and woody plants presents difficulties, limitations, and challenges. For instance, the occurrence of a tree disease can extend over successive growing seasons, not just during a specific growing season, which can impact the effectiveness of biocontrol strategies. As a result, the implementation of BCAs for disease control in trees and woody plants has been comparatively less extensive than their use in annual crops and herbaceous species. Despite this, there are many cases where biocontrol applications have been successfully used to improve forest plantation health and have even facilitated the emergence of commercial biocontrol products. One example is Dutch Trig^®^, which has been used for around 30 years to prevent elm trees from being infected by the blue stain fungus *O. novo-ulmi*, which is known to cause Dutch elm disease [189]. In order to enhance the effectiveness of applying BCAs in mitigating emerging forest diseases, more research is needed to unravel the dynamics of such diseases, how they interact with BCA measures, and what impact they may have on the ecology of a forested environment. Moreover, biological control with the application of BCAs must be combined with other measures (e.g., cultural practices, sanitary cuttings, tree breeding for disease resistance, and increasing tree species diversity) in an integrated disease management approach.

## Data Availability

All data are available in individual cited studies in this review.

## References

[1] Bettenfeld P., Fontaine F., Trouvelot S., Fernandez O., Courty P.-E. (2020). Woody plant declines. What’s wrong with the microbiome?. Trends Plant Sci..

[2] Brasier C.M. (2001). Rapid evolution of introduced plant pathogens via interspecific hybridization: Hybridization is leading to rapid evolution of Dutch elm disease and other fungal plant pathogens. Bioscience.

[3] Vettraino A., Morel O., Perlerou C., Robin C., Diamandis S., Vannini A. (2005). Occurrence and distribution of *Phytophthora* species in European chestnut stands, and their association with Ink Disease and crown decline. Eur. J. Plant Pathol..

[4] Rigling D., Prospero S. (2018). *Cryphonectria parasitica*, the causal agent of chestnut blight: Invasion history, population biology and disease control. Mol. Plant Pathol..

[5] Grünwald N.J., Garbelotto M., Goss E.M., Heungens K., Prospero S. (2012). Emergence of the sudden oak death pathogen *Phytophthora ramorum*. Trends Microbiol..

[6] Futai K. (2013). Pine wood nematode, *Bursaphelenchus xylophilus*. Annu. Rev. Phytopathol..

[7] Harrington T.C., Yun H.Y., Lu S.-S., Goto H., Aghayeva D.N., Fraedrich S.W. (2011). Isolations from the redbay ambrosia beetle, *Xyleborus glabratus*, confirm that the laurel wilt pathogen, *Raffaelea lauricola*, originated in Asia. Mycologia.

[8] Sturrock R., Frankel S., Brown A., Hennon P., Kliejunas J., Lewis K., Worrall J., Woods A. (2011). Climate change and forest diseases. Plant Pathol..

[9] Prospero S., Cleary M. (2017). Effects of host variability on the spread of invasive forest diseases. Forests.

[10] Holdenrieder O., Pautasso M., Weisberg P.J., Lonsdale D. (2004). Tree diseases and landscape processes: The challenge of landscape pathology. Trends Ecol. Evol..

[11] Yang L.-N., Ren M., Zhan J. (2022). Modeling plant diseases under climate change: Evolutionary perspectives. Trends Plant Sci..

[12] Wang J., Jia H. (2016). Metagenome-wide association studies: Fine-mining the microbiome. Nat. Rev. Microbiol..

[13] Poudel R., Jumpponen A., Schlatter D.C., Paulitz T., Gardener B.M., Kinkel L.L., Garrett K. (2016). Microbiome networks: A systems framework for identifying candidate microbial assemblages for disease management. Phytopathology.

[14] Van Der Heijden M.G., Hartmann M. (2016). Networking in the plant microbiome. PLoS Biol..

[15] Babalola O.O. (2010). Beneficial bacteria of agricultural importance. Biotechnol. Lett..

[16] Bulgarelli D., Schlaeppi K., Spaepen S., Van Themaat E.V.L., Schulze-Lefert P. (2013). Structure and functions of the bacterial microbiota of plants. Annu. Rev. Plant Biol..

[17] Neu A.T., Allen E.E., Roy K. (2021). Defining and quantifying the core microbiome: Challenges and prospects. Proc. Natl. Acad. Sci. USA.

[18] Vorholt J.A., Vogel C., Carlström C.I., Müller D.B. (2017). Establishing causality: Opportunities of synthetic communities for plant microbiome research. Cell Host Microbe.

[19] Liu H., Brettell L.E., Qiu Z., Singh B.K. (2020). Microbiome-mediated stress resistance in plants. Trends Plant Sci..

[20] French E., Kaplan I., Iyer-Pascuzzi A., Nakatsu C.H., Enders L. (2021). Emerging strategies for precision microbiome management in diverse agroecosystems. Nat. Plants.

[21] Prospero S., Botella L., Santini A., Robin C. (2021). Biological control of emerging forest diseases: How can we move from dreams to reality?. For. Ecol. Manag..

[22] Cai S., Jia J., He C., Zeng L., Fang Y., Qiu G., Lan X., Su J., He X. (2022). Multi-omics of pine wood nematode pathogenicity associated with culturable associated microbiota through an artificial assembly approach. Front. Plant Sci..

[23] Corinne V., Bastien C., Emmanuelle J., Heidy S. (2021). Trees and Insects Have Microbiomes: Consequences for Forest Health and Management. Curr. For. Rep..

[24] Asiegbu F.O., Asiegbu F.O., Kovalchuk A. (2021). Chapter 22—Forest microbiome: Challenges and future perspectives. Forest Microbiology.

[25] Burke D.J., Hoke A.J., Koch J. (2020). The emergence of beech leaf disease in Ohio: Probing the plant microbiome in search of the cause. For. Pathol..

[26] Li F., Zi H., Sonne C., Li X. (2023). Microbiome sustains forest ecosystem functions across hierarchical scales. Eco-Environ. Health.

[27] Baldrian P. (2016). Forest microbiome: Diversity, complexity and dynamics. FEMS Microbiol. Rev..

[28] Asiegbu F.O., Kovalchuk A. (2021). Forest Microbiology: Volume 1: Tree Microbiome: Phyllosphere, Endosphere and Rhizosphere.

[29] Frank A.C., Saldierna Guzmán J.P., Shay J.E. (2017). Transmission of Bacterial Endophytes. Microorganisms.

[30] Kuzyakov Y. (2002). Factors affecting rhizosphere priming effects. J. Plant Nutr. Soil Sci..

[31] Hinsinger P., Bengough A.G., Vetterlein D., Young I.M. (2009). Rhizosphere: Biophysics, biogeochemistry and ecological relevance. Plant Soil.

[32] Hansen E.M. (2015). Phytophthora species emerging as pathogens of forest trees. Curr. For. Rep..

[33] Coutinho T.A., Wingfield M.J. (2017). *Ralstonia solanacearum* and *R. pseudosolanacearum* on *Eucalyptus*: Opportunists or Primary Pathogens?. Front. Plant Sci..

[34] Jiang Y., Li B., Liu P., Liao F., Weng Q., Chen Q. (2016). First report of bacterial wilt caused by *Ralstonia solanacearum* on fig trees in China. For. Pathol..

[35] Roux J., Wingfield M. (2009). *Ceratocystis* species: Emerging pathogens of non-native plantation *Eucalyptus* and *Acacia* species. South. For. J. For. Sci..

[36] Barnes I., Roux J., Wingfield B., O’Neill M., Wingfield M. (2003). *Ceratocystis fimbriata* infecting *Eucalyptus grandis* in Uruguay. Australas. Plant Pathol..

[37] Markakis E.A., Tjamos S.E., Antoniou P.P., Paplomatas E.J., Tjamos E.C. (2016). Biological control of Verticillium wilt of olive by *Paenibacillus alvei*, strain K165. BioControl.

[38] Wang Y., Wang Y., Tian C. (2013). Quantitative detection of pathogen DNA of Verticillium wilt on smoke tree *Cotinus coggygria*. Plant Dis..

[39] Harris D., Hiemstra J. (1998). A Compendium of Verticillium Wilts in Tree Species.

[40] Hrynkiewicz K., Baum C. (2012). The Potential of Rhizosphere Microorganisms to Promote the Plant Growth in Disturbed Soils.

[41] Al-Karaki G.N., Shahid S., Taha F., Abdelfattah M. (2013). The role of mycorrhiza in the reclamation of degraded lands in arid environments. Developments in Soil Classification, Land Use Planning and Policy Implications.

[42] Khabou W., Hajji B., Zouari M., Rigane H., Abdallah F.B. (2014). Arbuscular mycorrhizal fungi improve growth and mineral uptake of olive tree under gypsum substrate. Ecol. Eng..

[43] Manaut N., Sanguin H., Ouahmane L., Bressan M., Thioulouse J., Baudoin E., Galiana A., Hafidi M., Prin Y., Duponnois R. (2015). Potentialities of ecological engineering strategy based on native arbuscular mycorrhizal community for improving afforestation programs with carob trees in degraded environments. Ecol. Eng..

[44] Kerr A. (2016). Biological control of crown gall. Australas. Plant Pathol..

[45] Prieto P., Navarro-Raya C., Valverde-Corredor A., Amyotte S.G., Dobinson K.F., Mercado-Blanco J. (2009). Colonization process of olive tissues by *Verticillium dahliae* and its in planta interaction with the biocontrol root endophyte *Pseudomonas fluorescens* PICF7. Microb. Biotechnol..

[46] Martínez-García P.M., Ruano-Rosa D., Schilirò E., Prieto P., Ramos C., Rodríguez-Palenzuela P., Mercado-Blanco J. (2015). Complete genome sequence of *Pseudomonas fluorescens* strain PICF7, an indigenous root endophyte from olive (*Olea europaea* L.) and effective biocontrol agent against *Verticillium dahliae*. Stand. Genom. Sci..

[47] Yi H.S., Yang J.W., Ryu C.M. (2013). ISR meets SAR outside: Additive action of the endophyte *Bacillus pumilus* INR7 and the chemical inducer, benzothiadiazole, on induced resistance against bacterial spot in field-grown pepper. Front. Plant Sci..

[48] Bais H.P., Weir T.L., Perry L.G., Gilroy S., Vivanco J.M. (2006). The role of root exudates in rhizosphere interactions with plants and other organisms. Annu. Rev. Plant Biol..

[49] Badri D.V., Vivanco J.M. (2009). Regulation and function of root exudates. Plant Cell Environ..

[50] Sasse J., Martinoia E., Northen T. (2018). Feed your friends: Do plant exudates shape the root microbiome?. Trends Plant Sci..

[51] Zhalnina K., Louie K.B., Hao Z., Mansoori N., da Rocha U.N., Shi S., Cho H., Karaoz U., Loqué D., Bowen B.P. (2018). Dynamic root exudate chemistry and microbial substrate preferences drive patterns in rhizosphere microbial community assembly. Nat. Microbiol..

[52] Hu L., Robert C.A., Cadot S., Zhang X., Ye M., Li B., Manzo D., Chervet N., Steinger T., Van Der Heijden M.G. (2018). Root exudate metabolites drive plant-soil feedbacks on growth and defense by shaping the rhizosphere microbiota. Nat. Commun..

[53] Baetz U., Martinoia E. (2014). Root exudates: The hidden part of plant defense. Trends Plant Sci..

[54] Rudrappa T., Czymmek K.J., Paré P.W., Bais H.P. (2008). Root-secreted malic acid recruits beneficial soil bacteria. Plant Physiol..

[55] Lebeis S.L., Paredes S.H., Lundberg D.S., Breakfield N., Gehring J., McDonald M., Malfatti S., Glavina del Rio T., Jones C.D., Tringe S.G. (2015). Salicylic acid modulates colonization of the root microbiome by specific bacterial taxa. Science.

[56] Zhang N., Wang D., Liu Y., Li S., Shen Q., Zhang R. (2014). Effects of different plant root exudates and their organic acid components on chemotaxis, biofilm formation and colonization by beneficial rhizosphere-associated bacterial strains. Plant Soil.

[57] Tian B., Pei Y., Huang W., Ding J., Siemann E. (2021). Increasing flavonoid concentrations in root exudates enhance associations between arbuscular mycorrhizal fungi and an invasive plant. ISME J..

[58] Bai B., Liu W., Qiu X., Zhang J., Zhang J., Bai Y. (2022). The root microbiome: Community assembly and its contributions to plant fitness. J. Integr. Plant Biol..

[59] Pérez-Jaramillo J.E., Mendes R., Raaijmakers J.M. (2016). Impact of plant domestication on rhizosphere microbiome assembly and functions. Plant Mol. Biol..

[60] Chaparro J.M., Badri D.V., Vivanco J.M. (2014). Rhizosphere microbiome assemblage is affected by plant development. ISME J..

[61] Warmink J.A., Nazir R., Corten B., van Elsas J.D. (2011). Hitchhikers on the fungal highway: The helper effect for bacterial migration via fungal hyphae. Soil Biol. Biochem..

[62] Kohlmeier S., Smits T.H.M., Ford R.M., Keel C., Harms H., Wick L.Y. (2005). Taking the fungal highway: Mobilization of pollutant-degrading bacteria by fungi. Environ. Sci. Technol..

[63] Barriuso J., Ramos Solano B., Fray R.G., Cámara M., Hartmann A., Gutiérrez Mañero F.J. (2008). Transgenic tomato plants alter quorum sensing in plant growth-promoting rhizobacteria. Plant Biotechnol. J..

[64] Schenk S.T., Schikora A. (2015). AHL-priming functions via oxylipin and salicylic acid. Front. Plant Sci..

[65] Bitas V., Kim H.-S., Bennett J.W., Kang S. (2013). Sniffing on microbes: Diverse roles of microbial volatile organic compounds in plant health. Mol. Plant Microbe Interact..

[66] Schmidt R., Cordovez V., De Boer W., Raaijmakers J., Garbeva P. (2015). Volatile affairs in microbial interactions. ISME J..

[67] Audrain B., Farag M.A., Ryu C.-M., Ghigo J.-M. (2015). Role of bacterial volatile compounds in bacterial biology. FEMS Microbiol. Rev..

[68] Cosetta C.M., Wolfe B.E. (2019). Causes and consequences of biotic interactions within microbiomes. Curr. Opin. Microbiol..

[69] Li P.-D., Zhu Z.-R., Zhang Y., Xu J., Wang H., Wang Z., Li H. (2022). The phyllosphere microbiome shifts toward combating melanose pathogen. Microbiome.

[70] Rolfe S.A., Griffiths J., Ton J. (2019). Crying out for help with root exudates: Adaptive mechanisms by which stressed plants assemble health-promoting soil microbiomes. Curr. Opin. Microbiol..

[71] Carrión V.J., Perez-Jaramillo J., Cordovez V., Tracanna V., De Hollander M., Ruiz-Buck D., Mendes L.W., van Ijcken W.F., Gomez-Exposito R., Elsayed S.S. (2019). Pathogen-induced activation of disease-suppressive functions in the endophytic root microbiome. Science.

[72] Kokalis-Burelle N., McSorley R., Wang K.-H., Saha S.K., McGovern R.J. (2017). Rhizosphere microorganisms affected by soil solarization and cover cropping in *Capsicum annuum* and *Phaseolus lunatus* agroecosystems. Appl. Soil Ecol..

[73] Smalla K., Wieland G., Buchner A., Zock A., Parzy J., Kaiser S., Roskot N., Heuer H., Berg G. (2001). Bulk and rhizosphere soil bacterial communities studied by denaturing gradient gel electrophoresis: Plant-dependent enrichment and seasonal shifts revealed. Appl. Environ. Microbiol..

[74] Mercado-Blanco J., Abrantes I., Barra Caracciolo A., Bevivino A., Ciancio A., Grenni P., Hrynkiewicz K., Kredics L., Proença D.N. (2018). Belowground microbiota and the health of tree crops. Front. Microbiol..

[75] Ab Rahman S.F.S., Singh E., Pieterse C.M., Schenk P.M. (2018). Emerging microbial biocontrol strategies for plant pathogens. Plant Sci..

[76] Trivedi P., Leach J.E., Tringe S.G., Sa T., Singh B.K. (2020). Plant–microbiome interactions: From community assembly to plant health. Nat. Rev. Microbiol..

[77] Yu L., Zi H., Zhu H., Liao Y., Li X. (2022). Rhizosphere microbiome of forest trees determines their resistance to soil-borne pathogens. Res. Sq..

[78] Vorholt J.A. (2012). Microbial life in the phyllosphere. Nat. Rev. Microbiol..

[79] Lindow S.E., Brandl M.T. (2003). Microbiology of the phyllosphere. Appl. Environ. Microbiol..

[80] Stone B.W., Weingarten E.A., Jackson C.R. (2018). The role of the phyllosphere microbiome in plant health and function. Annu. Plant Rev. Online.

[81] Sapp M., Ploch S., Fiore-Donno A.M., Bonkowski M., Rose L.E. (2018). Protists are an integral part of the *Arabidopsis thaliana* microbiome. Environ. Microbiol..

[82] Balogh B., Nga N.T.T., Jones J.B. (2018). Relative level of bacteriophage multiplication in vitro or in phyllosphere may not predict in planta efficacy for controlling bacterial leaf spot on tomato caused by *Xanthomonas perforans*. Front. Microbiol..

[83] Ritpitakphong U., Falquet L., Vimoltust A., Berger A., Métraux J.P., L’Haridon F. (2016). The microbiome of the leaf surface of *Arabidopsis* protects against a fungal pathogen. New Phytol..

[84] Xiong C., Zhu Y.G., Wang J.T., Singh B., Han L.L., Shen J.P., Li P.P., Wang G.B., Wu C.F., Ge A.H. (2021). Host selection shapes crop microbiome assembly and network complexity. New Phytol..

[85] Bodenhausen N., Horton M.W., Bergelson J. (2013). Bacterial communities associated with the leaves and the roots of *Arabidopsis thaliana*. PLoS ONE.

[86] Maignien L., DeForce E.A., Chafee M.E., Eren A.M., Simmons S.L. (2014). Ecological succession and stochastic variation in the assembly of *Arabidopsis thaliana* phyllosphere communities. mBio.

[87] Bai Y., Müller D.B., Srinivas G., Garrido-Oter R., Potthoff E., Rott M., Dombrowski N., Münch P.C., Spaepen S., Remus-Emsermann M. (2015). Functional overlap of the *Arabidopsis* leaf and root microbiota. Nature.

[88] Müller T., Ruppel S. (2014). Progress in cultivation-independent phyllosphere microbiology. FEMS Microbiol. Ecol..

[89] Peiffer J.A., Spor A., Koren O., Jin Z., Tringe S.G., Dangl J.L., Buckler E.S., Ley R.E. (2013). Diversity and heritability of the maize rhizosphere microbiome under field conditions. Proc. Natl. Acad. Sci. USA.

[90] Delmotte N., Knief C., Chaffron S., Innerebner G., Roschitzki B., Schlapbach R., von Mering C., Vorholt J.A. (2009). Community proteogenomics reveals insights into the physiology of phyllosphere bacteria. Proc. Natl. Acad. Sci. USA.

[91] Knief C., Delmotte N., Chaffron S., Stark M., Innerebner G., Wassmann R., Von Mering C., Vorholt J.A. (2012). Metaproteogenomic analysis of microbial communities in the phyllosphere and rhizosphere of rice. ISME J..

[92] Izhaki I., Fridman S., Gerchman Y., Halpern M. (2013). Variability of bacterial community composition on leaves between and within plant species. Curr. Microbiol..

[93] Inácio J., Pereira P., Carvalho M., Fonseca Á., Amaral-Collaço M., Spencer-Martins I. (2002). Estimation and diversity of phylloplane mycobiota on selected plants in a mediterranean–type ecosystem in Portugal. Microb. Ecol..

[94] Kembel S.W., O’Connor T.K., Arnold H.K., Hubbell S.P., Wright S.J., Green J.L. (2014). Relationships between phyllosphere bacterial communities and plant functional traits in a neotropical forest. Proc. Natl. Acad. Sci. USA.

[95] Griffiths S.M., Galambao M., Rowntree J., Goodhead I., Hall J., O’Brien D., Atkinson N., Antwis R.E. (2020). Complex associations between cross-kingdom microbial endophytes and host genotype in ash dieback disease dynamics. J. Ecol..

[96] Ulrich K., Becker R., Behrendt U., Kube M., Ulrich A. (2020). A comparative analysis of ash leaf-colonizing bacterial communities identifies putative antagonists of Hymenoscyphus fraxineus. Front. Microbiol..

[97] Durand A., Maillard F., Alvarez-Lopez V., Guinchard S., Bertheau C., Valot B., Blaudez D., Chalot M. (2018). Bacterial diversity associated with poplar trees grown on a Hg-contaminated site: Community characterization and isolation of Hg-resistant plant growth-promoting bacteria. Sci. Total Environ..

[98] Redford A.J., Bowers R.M., Knight R., Linhart Y., Fierer N. (2010). The ecology of the phyllosphere: Geographic and phylogenetic variability in the distribution of bacteria on tree leaves. Environ. Microbiol..

[99] Leff J.W., Jones S.E., Prober S.M., Barberán A., Borer E.T., Firn J.L., Harpole W.S., Hobbie S.E., Hofmockel K.S., Knops J.M. (2015). Consistent responses of soil microbial communities to elevated nutrient inputs in grasslands across the globe. Proc. Natl. Acad. Sci. USA.

[100] Zarraonaindia I., Owens S.M., Weisenhorn P., West K., Hampton-Marcell J., Lax S., Bokulich N.A., Mills D.A., Martin G., Taghavi S. (2015). The soil microbiome influences grapevine-associated microbiota. mBio.

[101] Copeland J.K., Yuan L., Layeghifard M., Wang P.W., Guttman D.S. (2015). Seasonal community succession of the phyllosphere microbiome. Mol. Plant Microbe Interact..

[102] Finkel O.M., Burch A.Y., Lindow S.E., Post A.F., Belkin S. (2011). Geographical location determines the population structure in phyllosphere microbial communities of a salt-excreting desert tree. Appl. Environ. Microbiol..

[103] Finkel O.M., Burch A.Y., Elad T., Huse S.M., Lindow S.E., Post A.F., Belkin S. (2012). Distance-decay relationships partially determine diversity patterns of phyllosphere bacteria on *Tamrix* trees across the Sonoran Desert. Appl. Environ. Microbiol..

[104] Laforest-Lapointe I., Messier C., Kembel S.W. (2016). Host species identity, site and time drive temperate tree phyllosphere bacterial community structure. Microbiome.

[105] Cordier T., Robin C., Capdevielle X., Desprez-Loustau M.-L., Vacher C. (2012). Spatial variability of phyllosphere fungal assemblages: Genetic distance predominates over geographic distance in a European beech stand (*Fagus sylvatica*). Fungal Ecol..

[106] Arnold A.E., Maynard Z., Gilbert G.S., Coley P.D., Kursar T.A. (2000). Are tropical fungal endophytes hyperdiverse?. Ecol. Lett..

[107] Rana K.L., Kour D., Sheikh I., Dhiman A., Yadav N., Yadav A.N., Rastegari A.A., Singh K., Saxena A.K. (2019). Endophytic fungi: Biodiversity, ecological significance, and potential industrial applications. Recent Advancement in White Biotechnology through Fungi.

[108] Perreault R., Laforest-Lapointe I. (2022). Plant-microbe interactions in the phyllosphere: Facing challenges of the anthropocene. ISME J..

[109] Zhu Y.G., Xiong C., Wei Z., Chen Q.L., Ma B., Zhou S.Y.D., Tan J., Zhang L.M., Cui H.L., Duan G.L. (2022). Impacts of global change on the phyllosphere microbiome. New Phytol..

[110] Jumpponen A., Jones K. (2009). Massively parallel 454 sequencing indicates hyperdiverse fungal communities in temperate *Quercus macrocarpa* phyllosphere. New Phytol..

[111] Balint M., Bartha L., O’Hara R.B., Olson M.S., Otte J., Pfenninger M., Robertson A.L., Tiffin P., Schmitt I. (2015). Relocation, high-latitude warming and host genetic identity shape the foliar fungal microbiome of poplars. Mol. Ecol..

[112] Balint M., Tiffin P., Hallstroem B., O’Hara R.B., Olson M.S., Fankhauser J.D., Piepenbring M., Schmitt I. (2013). Host genotype shapes the foliar fungal microbiome of balsam poplar (*Populus balsamifera*). PLoS ONE.

[113] Levy A., Salas Gonzalez I., Mittelviefhaus M., Clingenpeel S., Herrera Paredes S., Miao J., Wang K., Devescovi G., Stillman K., Monteiro F. (2018). Genomic features of bacterial adaptation to plants. Nat. Genet..

[114] Junker R.R., Tholl D. (2013). Volatile organic compound mediated interactions at the plant-microbe interface. J. Chem. Ecol..

[115] Cosoveanu A., Gimenez-Mariño C., Cabrera Y., Hernandez G., Cabrera R. (2014). Endophytic fungi from grapevine cultivars in Canary Islands and their activity against phytopatogenic fungi. Int. J. Agric. Crop Sci..

[116] Balint-Kurti P., Simmons S.J., Blum J.E., Ballaré C.L., Stapleton A.E. (2010). Maize leaf epiphytic bacteria diversity patterns are genetically correlated with resistance to fungal pathogen infection. Mol. Plant Microbe Interact..

[117] Golinska P., Wypij M., Agarkar G., Rathod D., Dahm H., Rai M. (2015). Endophytic actinobacteria of medicinal plants: Diversity and bioactivity. Antonie Van Leeuwenhoek.

[118] Thapa S., Prasanna R. (2018). Prospecting the characteristics and significance of the phyllosphere microbiome. Ann. Microbiol..

[119] Ali B., Sabri A., Ljung K., Hasnain S. (2009). Auxin production by plant associated bacteria: Impact on endogenous IAA content and growth of *Triticum aestivum* L.. Lett. Appl. Microbiol..

[120] Burch A.Y., Zeisler V., Yokota K., Schreiber L., Lindow S.E. (2014). The hygroscopic biosurfactant syringafactin produced by *Pseudomonas syringae* enhances fitness on leaf surfaces during fluctuating humidity. Environ. Microbiol..

[121] Hardoim P.R., Van Overbeek L.S., Berg G., Pirttilä A.M., Compant S., Campisano A., Döring M., Sessitsch A. (2015). The hidden world within plants: Ecological and evolutionary considerations for defining functioning of microbial endophytes. Microbiol. Mol. Biol. Rev..

[122] Chen T., Nomura K., Wang X., Sohrabi R., Xu J., Yao L., Paasch B.C., Ma L., Kremer J., Cheng Y. (2020). A plant genetic network for preventing dysbiosis in the phyllosphere. Nature.

[123] Bakker P.A., Pieterse C.M., de Jonge R., Berendsen R.L. (2018). The soil-borne legacy. Cell.

[124] Liu H., Li J., Carvalhais L.C., Percy C.D., Prakash Verma J., Schenk P.M., Singh B.K. (2021). Evidence for the plant recruitment of beneficial microbes to suppress soil-borne pathogens. New Phytol..

[125] Cordovez V., Dini-Andreote F., Carrión V.J., Raaijmakers J.M. (2019). Ecology and evolution of plant microbiomes. Annu. Rev. Microbiol..

[126] Getzke F., Thiergart T., Hacquard S. (2019). Contribution of bacterial-fungal balance to plant and animal health. Curr. Opin. Microbiol..

[127] Yin C., Casa Vargas J.M., Schlatter D.C., Hagerty C.H., Hulbert S.H., Paulitz T.C. (2021). Rhizosphere community selection reveals bacteria associated with reduced root disease. Microbiome.

[128] Kwak M.-J., Kong H.G., Choi K., Kwon S.-K., Song J.Y., Lee J., Lee P.A., Choi S.Y., Seo M., Lee H.J. (2018). Rhizosphere microbiome structure alters to enable wilt resistance in tomato. Nat. Biotechnol..

[129] Berg M., Koskella B. (2018). Nutrient-and dose-dependent microbiome-mediated protection against a plant pathogen. Curr. Biol..

[130] Ginnan N.A., Dang T., Bodaghi S., Ruegger P.M., McCollum G., England G., Vidalakis G., Borneman J., Rolshausen P.E., Roper M.C. (2020). Disease-induced microbial shifts in citrus indicate microbiome-derived responses to huanglongbing across the disease severity spectrum. Phytobiomes J..

[131] Jakuschkin B., Fievet V., Schwaller L., Fort T., Robin C., Vacher C. (2016). Deciphering the pathobiome: Intra-and interkingdom interactions involving the pathogen *Erysiphe alphitoides*. Microb. Ecol..

[132] Brader G., Compant S., Vescio K., Mitter B., Trognitz F., Ma L.-J., Sessitsch A. (2017). Ecology and genomic insights into plant-pathogenic and plant-nonpathogenic endophytes. Annu. Rev. Phytopathol..

[133] Baiyee B., Pornsuriya C., Ito S.-i., Sunpapao A. (2019). *Trichoderma spirale* T76-1 displays biocontrol activity against leaf spot on lettuce (*Lactuca sativa* L.) caused by *Corynespora cassiicola* or *Curvularia aeria*. Biol. Control..

[134] Loc N.H., Huy N.D., Quang H.T., Lan T.T., Thu Ha T.T. (2020). Characterisation and antifungal activity of extracellular chitinase from a biocontrol fungus, *Trichoderma asperellum* PQ34. Mycology.

[135] Singh M., Kumar A., Singh R., Pandey K.D. (2017). Endophytic bacteria: A new source of bioactive compounds. 3 Biotech.

[136] Yassin M.T., Mostafa A.A.-F., Al-Askar A.A., Sayed S.R., Rady A.M. (2021). Antagonistic activity of *Trichoderma harzianum* and *Trichoderma viride* strains against some fusarial pathogens causing stalk rot disease of maize, in vitro. J. King Saud Univ. Sci..

[137] Yan L., Khan R.A.A. (2021). Biological control of bacterial wilt in tomato through the metabolites produced by the biocontrol fungus, *Trichoderma harzianum*. Egypt. J. Biol. Pest Control.

[138] Kawaguchi A., Nita M., Ishii T., Watanabe M., Noutoshi Y. (2019). Biological control agent *Rhizobium* (=*Agrobacterium*) vitis strain ARK-1 suppresses expression of the essential and non-essential *vir* genes of tumorigenic *R. vitis*. BMC Res. Notes.

[139] Hu Z., Tao Y., Tao X., Su Q., Cai J., Qin C., Ding W., Li C. (2019). Sesquiterpenes with phytopathogenic fungi inhibitory activities from fungus *Trichoderma virens* from *Litchi chinensis* Sonn. J. Agric. Food Chem..

[140] Mengistu A.A. (2020). Endophytes: Colonization, behaviour, and their role in defense mechanism. Int. J. Microbiol..

[141] Mejía L.C., Herre E.A., Sparks J.P., Winter K., García M.N., Van Bael S.A., Stitt J., Shi Z., Zhang Y., Guiltinan M.J. (2014). Pervasive effects of a dominant foliar endophytic fungus on host genetic and phenotypic expression in a tropical tree. Front. Microbiol..

[142] Li Y., Duan T., Nan Z., Li Y. (2021). Arbuscular mycorrhizal fungus alleviates alfalfa leaf spots caused by *Phoma medicaginis* revealed by RNA-seq analysis. J. Appl. Microbiol..

[143] Busby P.E., Peay K.G., Newcombe G. (2016). Common foliar fungi of Populus trichocarpa modify Melampsora rust disease severity. New Phytol..

[144] Ren J.H., Ye J.R., Liu H., Xu X.L., Wu X.Q. (2011). Isolation and characterization of a new *Burkholderia pyrrocinia* strain JK-SH007 as a potential biocontrol agent. World J. Microbiol. Biotechnol..

[145] Doty S.L., Joubert P.M., Firrincieli A., Sher A.W., Tournay R., Kill C., Parikh S.S., Okubara P. (2023). Potential Biocontrol Activities of Populus Endophytes against Several Plant Pathogens Using Different Inhibitory Mechanisms. Pathogens.

[146] Kandel S.L., Firrincieli A., Joubert P.M., Okubara P.A., Leston N.D., McGeorge K.M., Mugnozza G.S., Harfouche A., Kim S.-H., Doty S.L. (2017). An In vitro Study of Bio-Control and Plant Growth Promotion Potential of Salicaceae Endophytes. Front. Microbiol..

[147] Tanney J.B., McMullin D.R., Green B.D., Miller J.D., Seifert K.A. (2016). Production of antifungal and antiinsectan metabolites by the *Picea* endophyte *Diaporthe maritima* sp. nov. Fungal Biol..

[148] Sumarah M.W., Kesting J.R., Sørensen D., Miller J.D. (2011). Antifungal metabolites from fungal endophytes of *Pinus strobus*. Phytochemistry.

[149] Sumarah M.W., Walker A.K., Seifert K.A., Todorov A., Miller J.D. (2015). Screening of fungal endophytes isolated from eastern white pine needles. The Formation, Structure and Activity of Phytochemicals.

[150] Segaran G., Sathiavelu M. (2019). Fungal endophytes: A potent biocontrol agent and a bioactive metabolites reservoir. Biocatal. Agric. Biotechnol..

[151] Zhou F., Emonet A., Dénervaud Tendon V., Marhavy P., Wu D., Lahaye T., Geldner N. (2020). Co-incidence of damage and microbial patterns controls localized immune responses in roots. Cell.

[152] Hacquard S., Spaepen S., Garrido-Oter R., Schulze-Lefert P. (2017). Interplay between innate immunity and the plant microbiota. Annu. Rev. Phytopathol..

[153] Zhang C., He J., Dai H., Wang G., Zhang X., Wang C., Shi J., Chen X., Wang D., Wang E. (2021). Discriminating symbiosis and immunity signals by receptor competition in rice. Proc. Natl. Acad. Sci. USA.

[154] Berendsen R.L., Pieterse C.M., Bakker P.A. (2012). The rhizosphere microbiome and plant health. Trends Plant Sci..

[155] Bakker P.A., Berendsen R.L., Van Pelt J.A., Vismans G., Yu K., Li E., Van Bentum S., Poppeliers S.W., Gil J.J.S., Zhang H. (2020). The soil-borne identity and microbiome-assisted agriculture: Looking back to the future. Mol. Plant..

[156] Conrath U., Beckers G.J., Flors V., García-Agustín P., Jakab G., Mauch F., Newman M.-A., Pieterse C.M., Poinssot B., Pozo M.J. (2006). Priming: Getting ready for battle. Mol. Plant Microbe Interact..

[157] Stockwell V., Johnson K., Sugar D., Loper J. (2002). Antibiosis contributes to biological control of fire blight by *Pantoea agglomerans* strain Eh252 in orchards. Phytopathology.

[158] Pusey P., Stockwell V., Reardon C., Smits T., Duffy B. (2011). Antibiosis activity of *Pantoea agglomerans* biocontrol strain E325 against *Erwinia amylovora* on apple flower stigmas. Phytopathology.

[159] Xu Z., Shao J., Li B., Yan X., Shen Q., Zhang R. (2013). Contribution of bacillomycin D in *Bacillus amyloliquefaciens* SQR9 to antifungal activity and biofilm formation. Appl. Environ. Microbiol..

[160] Xu Z., Zhang R., Wang D., Qiu M., Feng H., Zhang N., Shen Q. (2014). Enhanced control of cucumber wilt disease by *Bacillus amyloliquefaciens* SQR9 by altering the regulation of its DegU phosphorylation. Appl. Environ. Microbiol..

[161] Wang B., Yuan J., Zhang J., Shen Z., Zhang M., Li R., Ruan Y., Shen Q. (2013). Effects of novel bioorganic fertilizer produced by *Bacillus amyloliquefaciens* W19 on antagonism of *Fusarium* wilt of banana. Biol. Fertil. Soils.

[162] Yuan J., Raza W., Shen Q., Huang Q. (2012). Antifungal activity of *Bacillus amyloliquefaciens* NJN-6 volatile compounds against *Fusarium oxysporum* f. sp. *cubense*. Appl. Environ. Microbiol..

[163] Piewngam P., Zheng Y., Nguyen T.H., Dickey S.W., Joo H.-S., Villaruz A.E., Glose K.A., Fisher E.L., Hunt R.L., Li B. (2018). Pathogen elimination by probiotic *Bacillus via* signalling interference. Nature.

[164] Tian B., Xie J., Fu Y., Cheng J., Li B., Chen T., Zhao Y., Gao Z., Yang P., Barbetti M.J. (2020). A cosmopolitan fungal pathogen of dicots adopts an endophytic lifestyle on cereal crops and protects them from major fungal diseases. ISME J..

[165] Zhang J., Miao Y., Rahimi M.J., Zhu H., Steindorff A., Schiessler S., Cai F., Pang G., Chenthamara K., Xu Y. (2019). Guttation capsules containing hydrogen peroxide: An evolutionarily conserved NADPH oxidase gains a role in wars between related fungi. Environ. Microbiol..

[166] Zhang J., Bayram Akcapinar G., Atanasova L., Rahimi M.J., Przylucka A., Yang D., Kubicek C.P., Zhang R., Shen Q., Druzhinina I.S. (2016). The neutral metallopeptidase NMP1 of *Trichoderma guizhouense* is required for mycotrophy and self-defence. Environ. Microbiol..

[167] Li Z., Bai X., Jiao S., Li Y., Li P., Yang Y., Zhang H., Wei G. (2021). A simplified synthetic community rescues *Astragalus mongholicus* from root rot disease by activating plant-induced systemic resistance. Microbiome.

[168] Berendsen R.L., Vismans G., Yu K., Song Y., De Jonge R., Burgman W.P., Burmølle M., Herschend J., Bakker P.A.H.M., Pieterse C.M.J. (2018). Disease-induced assemblage of a plant-beneficial bacterial consortium. ISME J..

[169] Yuan J., Zhao J., Wen T., Zhao M., Li R., Goossens P., Huang Q., Bai Y., Vivanco J.M., Kowalchuk G.A. (2018). Root exudates drive the soil-borne legacy of aboveground pathogen infection. Microbiome.

[170] Cox C., Garrett K., Bockus W. (2005). Meeting the challenge of disease management in perennial grain cropping systems. Renew. Agric. Food Syst..

[171] Mercado-Blanco J., JJ Lugtenberg B. (2014). Biotechnological applications of bacterial endophytes. Curr. Biotechnol..

[172] Pautasso M., Schlegel M., Holdenrieder O. (2015). Forest health in a changing world. Microb. Ecol..

[173] Mazzola M., Freilich S. (2017). Prospects for biological soilborne disease control: Application of indigenous versus synthetic microbiomes. Phytopathology.

[174] Sarma B.K., Yadav S.K., Singh S., Singh H.B. (2015). Microbial consortium-mediated plant defense against phytopathogens: Readdressing for enhancing efficacy. Soil Biol. Biochem..

[175] Woo S.L., Pepe O. (2018). Microbial consortia: Promising probiotics as plant biostimulants for sustainable agriculture. Front. Plant Sci..

[176] Izquierdo-García L.F., González-Almario A., Cotes A.M., Moreno-Velandia C.A. (2020). *Trichoderma virens* Gl006 and *Bacillus velezensis* Bs006: A compatible interaction controlling *Fusarium* wilt of cape gooseberry. Sci. Rep..

[177] Wong C.K.F., Saidi N.B., Vadamalai G., Teh C.Y., Zulperi D. (2019). Effect of bioformulations on the biocontrol efficacy, microbial viability and storage stability of a consortium of biocontrol agents against Fusarium wilt of banana. J. Appl. Microbiol..

[178] Hassani M.A., Durán P., Hacquard S. (2018). Microbial interactions within the plant holobiont. Microbiome.

[179] Santhanam R., Menezes R.C., Grabe V., Li D., Baldwin I.T., Groten K. (2019). A suite of complementary biocontrol traits allows a native consortium of root-associated bacteria to protect their host plant from a fungal sudden-wilt disease. Mol. Ecol..

[180] Liechty Z., Santos-Medellín C., Edwards J., Nguyen B., Mikhail D., Eason S., Phillips G., Sundaresan V. (2020). Comparative analysis of root microbiomes of rice cultivars with high and low methane emissions reveals differences in abundance of methanogenic archaea and putative upstream fermenters. mSystems.

[181] Wei Z., Yang T., Friman V.-P., Xu Y., Shen Q., Jousset A. (2015). Trophic network architecture of root-associated bacterial communities determines pathogen invasion and plant health. Nat. Commun..

[182] Hu J., Wei Z., Friman V.-P., Gu S.-H., Wang X.-F., Eisenhauer N., Yang T.-J., Ma J., Shen Q.-R., Xu Y.-C. (2016). Probiotic diversity enhances rhizosphere microbiome function and plant disease suppression. mBio.

[183] Gu S., Wei Z., Shao Z., Friman V.-P., Cao K., Yang T., Kramer J., Wang X., Li M., Mei X. (2020). Competition for iron drives phytopathogen control by natural rhizosphere microbiomes. Nat. Microbiol..

[184] Traxler M.F., Watrous J.D., Alexandrov T., Dorrestein P.C., Kolter R. (2013). Interspecies interactions stimulate diversification of the *Streptomyces coelicolor* secreted metabolome. mBio.

[185] Ola A.R.B., Thomy D., Lai D., Brötz-Oesterhelt H., Proksch P. (2013). Inducing secondary metabolite production by the endophytic fungus *Fusarium tricinctum* through coculture with *Bacillus subtilis*. J. Nat. Prod..

[186] Pishchany G., Mevers E., Ndousse-Fetter S., Horvath D.J., Paludo C.R., Silva-Junior E.A., Koren S., Skaar E.P., Clardy J., Kolter R. (2018). Amycomicin is a potent and specific antibiotic discovered with a targeted interaction screen. Proc. Natl. Acad. Sci. USA.

[187] Jain A., Singh S., Kumar Sarma B., Bahadur Singh H. (2012). Microbial consortium–mediated reprogramming of defence network in pea to enhance tolerance against Sclerotinia sclerotiorum. J. Appl. Microbiol..

[188] Alizadeh H., Behboudi K., Ahmadzadeh M., Javan-Nikkhah M., Zamioudis C., Pieterse C.M.J., Bakker P.A.H.M. (2013). Induced systemic resistance in cucumber and *Arabidopsis thaliana* by the combination of *Trichoderma harzianum* Tr6 and *Pseudomonas* sp. Ps14. Biol. Control.

[189] Postma J., Goossen-van de Geijn H. (2016). Twenty-four years of Dutch Trig^®^ application to control Dutch elm disease. BioControl.

